# Global DNA methylation and the association between metal exposure and chronic kidney disease

**DOI:** 10.3389/fpubh.2023.1104692

**Published:** 2023-05-25

**Authors:** Yu-Mei Hsueh, Wei-Jen Chen, Hui-Ling Lee, Ya-Li Huang, Horng-Sheng Shiue, Sheng-Lun Hsu, Hsi-Hsien Chen, Ying-Chin Lin

**Affiliations:** ^1^Department of Family Medicine, Wan Fang Hospital, Taipei Medical University, Taipei, Taiwan; ^2^Department of Public Health, School of Medicine, College of Medicine, Taipei Medical University, Taipei, Taiwan; ^3^Department of Medicine, Section of Epidemiology and Population Sciences, Baylor College of Medicine, Houston, TX, United States; ^4^Department of Chemistry, Fu Jen Catholic University, New Taipei City, Taiwan; ^5^Department of Chinese Medicine, College of Medicine, Chang Gung University, Taoyuan, Taiwan; ^6^Division of Nephrology, Department of Internal Medicine, School of Medicine, College of Medicine, Taipei Medical University, Taipei, Taiwan; ^7^Division of Nephrology, Department of Internal Medicine, Taipei Medical University Hospital, Taipei, Taiwan; ^8^Department of Family Medicine, School of Medicine, College of Medicine, Taipei Medical University, Taipei, Taiwan; ^9^Department of Geriatric Medicine, School of Medicine, College of Medicine, Taipei Medical University, Taipei, Taiwan

**Keywords:** 5-methyl-2-deoxycytidine, arsenic, cadmium, lead, selenium, chronic kidney disease

## Abstract

**Introduction:**

Prior studies indicate that exposure to metals may alter DNA methylation. Evidence also shows that global DNA methylation is associated with chronic kidney disease (CKD). This study aimed to examine the association between CKD and 5-methyl-2-deoxycytidine (5mdC, %), a marker of global DNA methylation, and to evaluate the interaction between metal exposures and 5mdC (%) on CKD. We also explored the mediation effect of 5mdC (%) on the association between metal exposures and renal function (i.e., estimated glomerular filtration rate, eGFR).

**Methods:**

A total of 218 CKD patients and 422 controls were recruited in this case–control study. 5mdC (%), concentrations of blood lead and cadmium, plasma selenium, and total urinary arsenic were measured. CKD cases were clinically defined among patients with eGFR <60 mL/min/1.73 m^2^ for at least 3 months and without hemodialysis. Odds ratio (OR) and 95% confidence interval (CI) were estimated by logistic regression models to examine the association between metal exposures, 5mdC (%), and CKD, adjusted for confounders. Multivariable linear regression models were used to examine associations between metal exposures, 5mdC (%), and eGFR.

**Results and Discussion:**

CKD cases compared to controls had 6.06-fold (95% CI: 3.11–11.81) higher odds of having high blood cadmium and high 5mdC (%) levels. A positive interaction on an additive scale was identified between blood cadmium and 5mdC (%) on CKD. Cases compared to controls had 4.73-fold (95% CI: 2.65–8.45) higher odds of having low plasma selenium and high 5mdC (%) levels; and a significant multiplicative interaction between plasma selenium and 5mdC (%) on CKD was observed. In addition, we found that blood lead and cadmium concentrations were positively associated, while plasma selenium concentrations were inversely associated, with 5mdC (%). The associations of blood lead and plasma selenium with eGFR were partially mediated by 5mdC (%). Our results suggest that 5mdC (%) may interact with plasma selenium and blood cadmium to influence the risk of CKD. The 5mdC (%) also potentially mediates the associations between exposure to metals and renal function.

## 1. Introduction

Chronic kidney disease (CKD) is a worldwide public health problem, with an estimated global mean prevalence of 13.4% (1). In Taiwan, the estimated prevalence rate of CKD is as high as 15.46%, and the incidence of CKD is 27.21/1,000 person-years (2). Moreover, end-stage renal disease is widespread in Taiwan, which leads to the highest incidence of renal dialysis therapy in the world (3). Therefore, CKD is a very important issue in Taiwan.

We previously found associations of increasing levels of total urinary arsenic and blood lead and cadmium with reduced estimated glomerular filtration rate (eGFR), while increasing levels of plasma selenium were associated with increasing eGFR (4, 5). Although our studies provided evidence of associations of exposure to arsenic, lead, cadmium, and selenium with CKD (4, 5), the biological mechanism underlying these associations remains unclear. One hypothesis mechanism is that chronic inflammation may cause abnormal DNA methylation and followed by causing glomerular and tubular interstitial lesions (6). DNA methylation is an epigenetic marker that promotes the transfer of methyl groups to cytosine, thereby affecting the expression of CpG rich genes that have an essential role in cell function and maintaining genomic stability (7). Evidence has shown that alteration of DNA methylation in peripheral immune cells has a profound impact on the development of diabetes-or hypertension-related CKD (8). Methylation of DNA cytosine (5-methyl-2-deoxycytidine, 5mdC), a recognized global DNA methylation marker, is the product of the base excision repair and nucleotide excision repair pathways of active DNA methylation (9) and is involved in genome imprinting, X chromosome inactivation, and gene expression regulation (10). Measuring the ratio of 5mdC to 2-deoxyguanine (dG) provides an accurate method for estimating total genomic DNA methylation levels (11, 12). 5mdC (%) is the best-characterized epigenetic modification in mammals (9). Thus, the use of 5mdC (%) may allow exploring whether global DNA methylation is associated with CKD.

Several studies to date have implicated exposure to metals in altering global DNA methylation. Low levels of lead exposure were associated with changes in DNA methylation related to neurodevelopment in a study among Peruvian adults who resided close to mining activities (13). Additionally, a Mexican study found that prenatal lead exposure was associated with global DNA methylation in adolescence (14). Long-term cadmium exposure reduces antioxidant capacity, causes oxidative damage to lipids and DNA, and reduces global DNA methylation levels in juvenile Nile tilapia (15). A review study summarized evidence from *in vitro*, animal, and human studies, and suggested an inverse association between selenium and global DNA methylation (16); however, another study recently found that selenium was positively associated with DNA methylation (17). Chronic arsenic exposure in parents leads to changes in global DNA methylation and transgenerational genotoxicity, which may be associated with reproductive defects in rats (18). A review paper has summarized environmental exposures, including air pollution, metals, persistent organic compounds, etc., that were associated with DNA methylation in the human population (19). In a recent systematic review encompassing epidemiologic studies and toxicologic animal evidence (20), Elkin et al. concluded that exposure to lead, arsenic, and cadmium was associated with DNA methylation signatures in adults, though replication testing is needed. As epigenetic signatures may have an important biologic effect of metal exposures, continued and expanded studies are needed to understand the role of global DNA methylation in the association with metal exposure-related diseases such as CKD.

The present study aimed to explore the role of global DNA methylation in the association between exposure to metals and CKD. We first evaluated the association of global DNA methylation, using 5mdC (%) as an indicator, with CKD. Secondly, we examined the interaction between exposure to metals and 5mdC (%) on CKD. Given a limited number of prior studies conducted interaction analysis between metal exposures and DNA methylation, both multiplicative and additive scales were conducted in our study to test for the interaction. In addition, we conducted a mediation analysis to explore the mediation effect of 5mdC (%) on the association between exposure to metals and renal function (i.e., eGFR).

## 2. Materials and methods

### 2.1. Study subjects

A clinic-based case–control study was conducted among clinically confirmed 218 CKD patients and 422 controls recruited from Taipei Medical University Hospital and Taipei Municipal Wan Fang Hospital (21). Frequency matching was employed in our study to balance the overall distributions of age and sex between cases and controls. Clinical confirmed CKD patients were recruited at the Department of Internal Medicine and Nephrology and diagnosed based on biochemical criteria, including blood urea nitrogen, proteinuria, and serum creatinine. eGFR (mL/min/1.73 m^2^) was calculated by using the equation from the Modified Diet Formula for Renal Disease: 186.3 × (serum creatinine)^-1.154^ × (age)^-0.203^ × 0.742 (if female) (22). According to the clinical practice guidelines from the Kidney Disease Outcomes Quality Initiative (23), patients with eGFR <60 mL/min/1.73 m^2^ for at least 3 months and without hemodialysis were classified as CKD. Healthy controls with no evidence of CKD were recruited from the Department of Family Medicine. All participants provided written informed consent before being recruited in the study. This study was approved by the Institutional Review Board of Taipei Medical University (N201912103), which was conducted in accordance with the Declaration of Helsinki.

### 2.2. Specimen collection and covariates

Peripheral blood samples were collected at enrollment in an EDTA-vacuum syringe and separated as plasma, red blood cells, and buffy coat. Urine samples were also collected in sterile polypropylene containers.

All participants completed an interview for the collection of data on sociodemographic characteristics, lifestyle, and disease histories (21). Covariates included sex, age (years), educational level (illiterate/elementary school, junior/senior high school, and college and above), cigarette smoking (non-smoker, former smoker, and current smoker), alcohol, tea, and coffee consumption (never/occasional or frequently), analgesic usage (no, as-needed basis, and routinely), and personal history of hypertension and diabetes (yes/no).

### 2.3. Metal exposures measurement

As described previously (5), blood lead and cadmium and plasma selenium were quantified by inductively coupled plasma mass spectrometry. Urinary arsenic species including arsenite (As^III^), arsenate (As^V^), monomethylarsonic acid (MMA^V^), and dimethylarsinic acid (DMA^V^), were measured by high-performance liquid chromatography-hydride generator-atomic absorption spectrometry (21). Concentrations of total urinary arsenic were defined as the sum of the concentrations of As^III^, As^V^, MMA^V^, and DMA^V^, and then divided by the urinary creatinine concentrations to adjust for hydration status (24). The detection limits, standard reference materials, and reliability of the metal measurements are shown in Supplementary Table S1.

### 2.4. Global DNA methylation markers: 5mdC measurement

Genomic DNA was extracted by digestion with proteinase K followed by phenol and chloroform. The levels of 5mdC were quantified by high-performance liquid chromatography (Agilent 1260VL, Agilent Technology, United States) equipped with an API 3000™ triple quadrupole mass spectrometer (AB SCIEX, Canada). The data were processed in the multiple reaction monitoring mode through Analyst1.4.2™ software (AB SCIEX). The detailed protocol of the analysis was described previously (11). The levels of 5mdC in the DNA sample were expressed as a percentage of the total cytosine content (methylated and non-methylated) using the following equation: 5mdC (%) = [(5mdC/dG) × 100] (9).

### 2.5. Statistical analysis

All data were analyzed using SAS 9.4 software (SAS Institute, Cary, NC, United States). A two-sided value of *p* < 0.05 was considered significant. Characteristics were summarized using the mean ± standard deviation for continuous variables and frequency (percentage) for categorical variables. The Wilcoxon rank-sum test was conducted to compare the metal concentrations and levels of 5mdC (%) between CKD cases and controls. Logistic regression models were used to evaluate the associations between metal exposures, 5mdC (%), and CKD by estimating odds ratio (OR) and 95% confidence interval (CI). The concentrations of metals and levels of 5mdC (%) were categorized into tertiles according to the distribution of the controls, and the lowest tertile was used as the reference group in the logistic regression models. Cochran–Armitage test was used for assessing the linear trend for ORs. All models included in interaction and mediation analyses were adjusted for frequency-matching variables (i.e., age and sex) and confounding. Confounding was informed by prior knowledge and met the criterion that was associated with CKD (value of *p* < 0.05; i.e., educational level, consumption of alcohol, coffee, and tea, analgesic usage, and personal history of hypertension and diabetes).

#### 2.5.1. Interaction analysis

To explore whether 5mdC (%) was an effect modifier of the association between exposure to metals and CKD, a product term combining the metal exposure and 5mdC (%) was added to the logistic regression models for testing multiplicative interaction. A value of *p* for interaction term <0.05 is indicative of a multiplicative interaction between metal exposures and 5mdC (%). We also examined the interaction between metal exposures and 5mdC (%) on CKD based on the additive scale. We classified the concentrations of metals and levels of 5mdC (%) based on their median levels according to the distribution of the controls and create a combined exposure variable [e.g., low lead/low 5mdC (%), low lead/high 5mdC (%), high lead/low 5mdC (%), and high lead/high 5mdC (%)]. This combined variable was assessed for its association with CKD in the logistic regression models. The synergy index (SI), relative excess risk due to interaction (RERI), and the attributable proportion (AP) were calculated to quantify additive interactions (25).

#### 2.5.2. Mediation analysis

Our study conducted the mediation analysis using linear regression models, estimated coefficient (β), and standard error (SE), to explore the mediation effect of 5mdC (%) on the association between exposure to metals and renal function (i.e., eGFR). The concentrations of metal, 5mdC (%), and eGFR were assessed as a continuous measure in the models. The test of mediating effect was conducted using the Sobel test (26). The path diagrams were shown to present the total effect [represents the effect of metals on eGFR without controlling for 5mdC (%)], direct effect [represents the effect of metals on eGFR after controlling for 5mdC (%)], and indirect effect [represents the effect of metals on eGFR through 5mdC (%)]. The proportion of mediation was estimated by the ratio of indirect effect to total effect (27).

## 3. Results

### 3.1. Participant characteristics

The sociodemographic characteristics, lifestyle, and disease histories between CKD cases and controls are shown in Table 1. The mean and standard deviation of age in the 218 CKD cases and 422 controls were 65.04 ± 13.46 and 64.26 ± 12.63 years, respectively. The CKD cases had a lower educational level compared to controls, with most cases either illiterate or having an elementary school education. No difference in smoking status was observed between cases and controls. More than one-third of controls reported having a habit of consuming alcohol occasionally or frequently compared to the cases. Additionally, over half of the controls reported consuming coffee and tea occasionally or frequently. Of cases, 12.39% reported routinely using analgesics, which was higher than controls. A higher percentage of cases had disease histories of diabetes and hypertension compared to controls.

**Table 1:** Sociodemographic characteristics, lifestyle, and disease histories between the CKD cases and controls.

Variables	CKD cases (*n* = 218)	Controls (*n* = 422)	Age-sex adjusted OR (95% CI)
Sex			
Male	134 (60.90)	257 (60.90)	1.00
Female	84 (39.10)	165 (39.10)	0.99 (0.71–1.39)^a^
Age	65.04 ± 13.46	64.26 ± 12.63	1.01 (0.99–1.02)^b^
Educational level			
Illiterate/elementary school	90 (41.28)	96 (22.75)	1.00^§^
Junior/senior high school	72 (32.03)	147 (34.83)	0.49 (0.32–0.74)^***^
College and above	56 (25.69)	179 (42.42)	0.30 (0.19–0.47)^***^
Cigarette smoking			
Non-smoker	160 (73.39)	307 (72.75)	1.00
Former smoker	33 (15.14)	72 (17.06)	0.85 (0.52–1.38)
Current smoker	25 (11.47)	43 (10.19)	1.13 (0.65–1.97)
Alcohol consumption			
Never	179 (82.11)	268 (63.51)	1.00
Occasional or frequently	39 (17.89)	154 (36.49)	0.34 (0.23–0.52)^***^
Coffee consumption			
Never	170 (77.98)	219 (51.90)	1.00
Occasional or frequently	48 (22.02)	203 (48.10)	0.30 (0.21–0.44)^***^
Tea consumption			
Never	122 (55.96)	149 (35.11)	1.00
Occasional or frequently	96 (44.04)	273 (64.69)	0.42 (0.30–0.59)^***^
Analgesic usage			
No	186 (85.32)	322 (76.30)	1.00
Yes, as-needed basis	5 (2.29)	81 (19.19)	0.11 (0.04–0.27)^***^
Yes, routinely	27 (12.39)	19 (4.50)	2.48 (1.34–4.59)^**^
Diabetes			
No	132 (60.55)	378 (89.57)	1.00
Yes	86 (39.45)	44 (10.43)	5.62 (3.71–8.52)^***^
Hypertension			
No	94 (43.12)	297 (70.38)	1.00
Yes	124 (56.88)	125 (29.60)	3.23 (2.28–4.58)^***^

CKD, chronic kidney disease; OR, odds ratio; CI, confidence interval.

Values expressed as mean ± standard deviation or number (%).

a Age-adjusted OR and 95% CI.

b Sex-adjusted OR and 95% CI.

^**^p < 0.01, ^***^p < 0.001, and ^§^*p* < 0.05 for trend test.

### 3.2. Associations between metal exposures, 5mdC (%), and CKD

Table 2 shows the associations between metal exposures, 5mdC (%), and CKD. In multivariable adjusted models, CKD cases compared to controls had 9.00-fold (95% CI: 4.83–16.75) higher odds of having blood lead levels >46.88 μg/dL than blood lead levels ≤28.68 μg/dL. Cases compared to controls had 10.36-fold (95% CI: 5.50–19.54) higher odds of having blood cadmium levels >1.30 μg/L than blood cadmium levels ≤0.82 μg/L. In contrast, cases compared to controls had 0.15-fold (95%CI: 0.08–0.26) lower odds of having plasma selenium levels >243.8 μg/L than plasma selenium levels ≤196.0 μg/L. The 5mdC (%) levels were higher in CKD cases compared to controls (mean ± standard deviation: 5.08 ± 2.51 vs. 4.23 ± 1.77). After adjusting for multivariable in the models, cases compared to controls had 2.14-fold (95%CI: 1.33–3.44) higher odds of having 5mdC levels >4.55% than 5mdC levels ≤3.60%.

**Table 2:** The associations between metal exposures, 5mdC (%), and CKD.

Variables	CKD cases (*n* = 218)	Controls (*n* = 422)	Age-sex adjusted OR (95% CI)	Multivariable adjusted OR (95% CI)^a^
Blood lead (μg/dL)	68.94 ± 38.95	42.50 ± 23.09 ^b^		
≤ 28.68	20 (9.17)	141 (33.41)	1.00 ^§^	1.00 ^§^
28.68–46.88	50 (22.94)	141 (33.41)	2.55 (1.44–4.53)^**^	2.89 (1.49–5.60)^**^
> 46.88	148 (67.89)	140 (33.18)	7.67 (4.52–13.30)^***^	9.00 (4.83–16.75)^***^
Blood cadmium (μg/L)	2.43 ± 3.46	1.24 ± 0.94 ^b^		
≤ 0.82	23 (10.55)	146 (34.60)	1.00 ^§^	1.00 ^§^
0.82–1.30	43 (19.72)	136 (32.23)	2.19 (1.24–3.86)^**^	2.49 (1.28–4.85)^**^
> 1.30	152 (69.72)	140 (33.18)	7.62 (4.56–12.73)^***^	10.36 (5.50–19.54)^***^
Plasma selenium (μg/L)	193.30 ± 69.79	218.50 ± 53.45 ^b^		
≤ 196.0	135 (61.93)	141 (33.41)	1.00 ^§^	1.00 ^§^
196.0–243.8	48 (22.02)	142 (33.65)	0.33 (0.22–0.50)^***^	0.23 (0.14–0.39)^***^
> 243.8	35 (16.06)	139 (32.94)	0.25 (0.16–0.39)^***^	0.15 (0.08–0.26)^***^
Total urinary arsenic (μg/g creatinine)	27.40 ± 21.76	20.02 ± 13.97 ^b^		
≤ 11.97	35 (16.06)	140 (33.18)	1.00 ^§^	1.00 ^§^
11.97–22.15	71 (32.57)	141 (33.41)	2.04 (1.28–3.27)^**^	2.11 (1.23–3.62)^**^
> 22.15	112 (51.38)	141 (33.41)	3.30 (2.09–5.21)^***^	3.43 (2.00–5.88)^***^
5mdC (%)	5.08 ± 2.51	4.23 ± 1.77 ^b^		
≤ 3.60	54 (24.77)	140 (33.18)	1.00 ^§^	1.00 ^§^
3.60–4.55	44 (20.18)	142 (33.65)	0.78 (0.49–1.23)	0.93 (0.54–1.61)
> 4.55	120 (55.05)	140 (33.18)	2.28 (1.53–3.39)^***^	2.14 (1.33–3.44)^***^

CKD, chronic kidney disease; 5mdC, 5-methyl-2-deoxycytidine; OR, odds ratio; CI, confidence interval.

Values expressed as mean ± standard deviation or number (%).

a Adjusted for age, sex, educational level, consumption of alcohol, coffee, and tea, analgesic usage, and personal history of hypertension and diabetes.

b *p* < 0.05 for comparison using Wilcoxon rank-sum test.

^**^p < 0.01, ^***^p < 0.001, and ^§^*p* < 0.05 for trend test.

### 3.3. The interaction between metal exposures and 5mdC (%) on CKD

Table 3 shows the combined effects of metal exposures and 5mdC (%) on CKD. After adjusting for multivariable in the models, cases compared to controls had 6.49-fold (95%CI: 3.41–12.36) higher odds of having blood lead levels >37.51 μg/dL and 5mdC levels >4.10% than blood lead levels ≤37.51 μg/dL and 5mdC levels ≤4.10%. Also, the ORs increased gradually from no risk factors [low levels of blood lead and 5mdC (%)], one risk factor [high levels of blood lead or 5mdC (%)], to two risk factors [high levels of blood lead and 5mdC (%)]. Similar associations were also observed in assessing the combined effect of blood cadmium and total urinary arsenic with 5mdC (%) on CKD. A significant positive interaction on an additive scale was identified between blood cadmium and 5mdC (%) on CKD [synergy index = 2.90 (1.13–7.45), RERI = 3.13 (0.62–6.00) and AP = 0.55 (0.29–0.81)], indicating that combined effect was more than the sum of the effects of having blood cadmium levels >1.30 μg/L and 5mdC levels >4.10%. We found that the odds of having plasma selenium levels ≤217.70 μg/L and 5mdC levels >4.10% were higher among CKD cases compared to controls [multivariable adjusted OR (95% CI) = 4.73 (2.65–8.45) relative to the low plasma selenium levels and high 5mdC (%) levels]. We observed a significant multiplicative interaction between plasma selenium and 5mdC (%) on CKD (interaction *p* < 0.001).

**Table 3:** The combined effect of metal exposures and 5mdC (%) on CKD.

Metal exposures	5mdC (%)	Case/Control (*n*)	Age-sex adjusted OR (95% CI)	Multivariable adjusted OR (95% CI)^a^
Blood lead (μg/dL)				
≤ 37.51	≤ 4.10	21/109	1.00 ^§^	1.00 ^§^
≤ 37.51	> 4.10	24/102	1.21 (0.63–2.32)	1.26 (0.60–2.65)
> 37.51	≤ 4.10	57/102	2.92 (1.65–5.17) ^***^	3.90 (1.97–7.69) ^***^
> 37.51	> 4.10	116/109	5.55 (3.25–9.50) ^***^	6.49 (3.41–12.36) ^***^
*p _interaction_*			0.53	0.79
Synergy index			2.14 (1.07–4.26)	1.74 (0.87–3.48)
RERI			2.42 (0.48–4.35)	2.34 (−0.45–5.13)
AP			0.44 (0.18–0.70)	0.36 (0.02–0.70)
Blood cadmium (μg/L)				
≤ 1.04	≤ 4.10	19/106	1.00 ^§^	1.00 ^§^
≤ 1.04	> 4.10	26/109	1.29 (0.67–2.48)	0.93 (0.44–1.99)
> 1.04	≤ 4.10	59/105	3.20 (1.78–5.75) ^***^	2.81 (1.40–5.64) ^***^
> 1.04	> 4.10	114/102	6.35 (3.63–11.10) ^***^	6.06 (3.11–11.81) ^***^
*p _interaction_*			0.50	0.15
Synergy index			2.15 (1.13–4.08)	2.90 (1.13–7.45)
RERI			2.86 (0.60–5.12)	3.13 (0.62–6.00)
AP			0.45 (0.20–0.70)	0.55 (0.29–0.81)
Plasma selenium (μg/L)				
> 217.70	≤ 4.10	31/114	1.00 ^§^	1.00 ^§^
> 217.70	> 4.10	30/97	1.16 (0.65–2.05)	0.89 (0.45–1.73)
≤ 217.70	≤ 4.10	47/97	1.83 (1.07–3.14)^*^	2.39 (1.26–4.53)^**^
≤ 217.70	> 4.10	110/114	3.67 (2.26–5.96)^***^	4.73 (2.65–8.45)^***^
*p _interaction_*			< 0.001	< 0.001
Synergy index			2.69 (0.90–8.11)	2.92 (0.94–9.12)
RERI			1.68 (0.40–2.96)	2.45 (0.40–4.51)
AP			0.46 (0.18–0.74)	0.52 (0.22–0.82)
Total urinary arsenic (μg/g creatinine)				
≤ 16.15	≤ 4.10	16/92	1.00 ^§^	1.00 ^§^
≤ 16.15	> 4.10	43/119	2.05 (1.08–3.89)^*^	2.38 (1.17–4.84)^*^
> 16.15	≤ 4.10	62/119	2.99 (1.62–5.52)^***^	3.56 (1.77–7.16)^***^
> 16.15	> 4.10	97/92	6.06 (3.31–11.07)^***^	5.73 (2.88–11.40)^***^
*p _interaction_*			0.46	0.94
Synergy index			1.67 (0.94–2.96)	1.20 (0.65–2.23)
RERI			2.02 (−0.15–4.19)	0.79 (−1.82–3.40)
AP			0.33 (0.05–0.62)	0.14 (−0.29–0.57)

CKD, chronic kidney disease; 5mdC, 5-methyl-2-deoxycytidine; OR, odds ratio; CI, confidence interval; *p*_interaction_, value of *p* for interaction product term; RERI, relative excess risk due to interaction; AP, attributable proportion.

a Adjusted for age, sex, educational level, consumption of alcohol, coffee, and tea, analgesic usage, and personal history of hypertension and diabetes.

^*^p < 0.05, ^**^p < 0.01, ^***^p < 0.001, and ^§^*p* < 0.05 for trend test.

### 3.4. Mediation effect of 5mdC (%) on the association between metal exposures and eGFR

**Figure 1:**
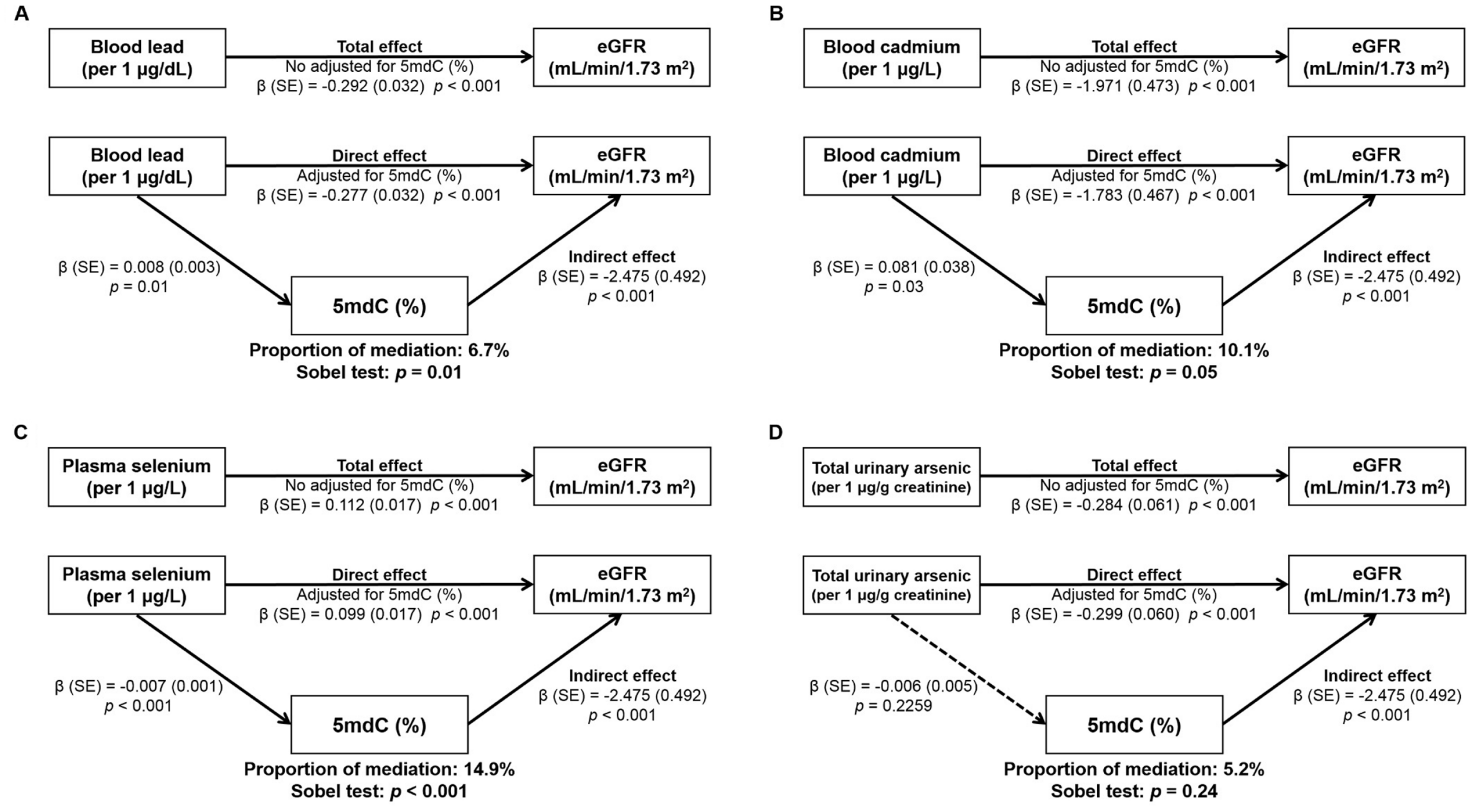
Mediation analysis of the estimated effect of exposure to metals on eGFR through 5mdC: **(A)** blood lead, **(B)** blood cadmium, **(C)** plasma selenium, and **(D)** total urinary arsenic. Effect estimates were adjusted for age, sex, educational level, consumption of alcohol, coffee, and tea, analgesic usage, and personal history of hypertension and diabetes.

The associations between exposure to metals, 5mdC (%), and eGFR are displayed in Figure 1. Increases in blood lead and cadmium and total urinary arsenic were each associated with a decrease of eGFR, while an increase of plasma selenium was associated with an increase of eGFR (see arrow of total effect in Figure 1). These associations remained significant after further adjusting 5mdC (%) in the models though the estimated effect was slightly attenuated (see arrow of direct effect in Figure 1). In addition, blood cadmium and lead were associated with increased 5mdC (%), while plasma selenium was associated with reduced 5mdC (%). However, we observed no association between total urinary arsenic and 5mdC (%). Additionally, an increase of 1% in 5mdC (%) was significantly associated with a decrease of 2.475 mL/min/1.73 m^2^ of eGFR (see arrow of indirect effect in Figure 1). In order to test whether 5mdC (%) may have mediated the associations between exposure to metals and CKD, the Sobel test was conducted among the observed associations of Figure 1. The mediating effect of 5mdC (%) was significant in the associations of blood lead (*p* < 0.01) and plasma selenium (*p* < 0.01) with eGFR. However, the test of the mediating effect for blood cadmium (*p* = 0.05) and total urinary arsenic (*p* = 0.24) was not significant. The 5mdC (%) mediated 6.7 and 14.9% of the associations of blood lead and plasma selenium, respectively, with eGFR in the mediation analysis.

## 4. Discussion

The present study added to the current knowledge of assessing associations between exposure to metals, 5mdC (%), and CKD. The higher odds of elevated 5mdC (%) were observed among CKD cases compared to controls. We also found a significant synergic effect of blood cadmium and 5mdC (%) on CKD; and a significant multiplicative interacted effect of plasma selenium and 5mdC (%) on CKD. In addition, we observed that blood lead and cadmium concentrations were significantly positively associated, but plasma selenium was significantly negatively associated, with 5mdC (%). Our study is the first to explore the mediating role of 5mdC (%) in the associations between exposure to metals and renal function (i.e., eGFR) and showed that 5mdC (%) might partially mediate the effects of blood lead and plasma selenium on eGFR.

Our study found that the odds of occasional or frequent alcohol, coffee, and tea consumption were lower in CKD cases than in controls, which is consistent with the results from other studies (28–30). These protective effects may be because coffee contains antioxidant and anti-inflammatory components (31), and alcohol and tea contain polyphenols that can reduce reactive oxygen species and anti-inflammatory components (32, 33). We also found that frequent analgesics use, hypertension, and diabetes were significantly associated with CKD, and these results were similar to other studies (34, 35).

Epigenetic modifications, including DNA methylation, are heritable patterns that modulate gene expression without altering DNA sequence (36), and have been implicated in the development of CKD (37). Thus, understanding the role of DNA methylation involved in the etiology of CKD may provide insights into the pathophysiology, severity, and prognosis of kidney disease (38). Evidence from *in vitro* and *in vivo* has supported that aberrant DNA methylation was associated with diabetic kidney disease and the progression of renal fibrosis (39, 40). While not focusing on global DNA methylation, studies have identified DNA hypomethylation and hypermethylation at specific loci among CKD patients (41) and found that specific DNA methylation was associated with a reduction in kidney function (42). In the present study, we observed that increased levels of 5mdC (%), a global DNA methylation indicator, were associated with decreased eGFR, and also CKD cases compared to controls had higher odds of having elevated 5mdC levels. This is consistent with a Japanese prospective study that recruited 308 CKD patients (43), which showed that concentrations of 5mdC (measured in urine specimens) were significantly higher among late-stage compared to early-to mid-stages patients, and increased 5mdC levels were associated with low eGFR (<30 mL/min/1.73m^2^; OR = 2.36, 95%CI: 1.24–4.60) in the multivariable adjusted model. However, in a cross-sectional study of 78 kidney disease patients among stages 2–4 conducted in the Netherlands (44), no differences were found between the levels of global DNA methylation (measured by the ratio of 5-methylcytosine and total cytosine ratio in the blood specimens) and kidney disease stages. Using different indicators of global DNA methylation and study design may be reasons for the inconsistencies observed between our study and the Netherlands’ study. Nevertheless, Ingrosso and Perna reviewed current evidence from *in vitro*, *in vivo*, and human studies regarding the role of epigenetic alterations in renal disease and suggested that DNA methylation, either global or gene-specific, may be a prospective biomarker of CKD (45).

Previous *in vitro* and *in vivo* studies have implicated that exposure to metals may alter DNA methylation. A study of cell-line experiments found that cadmium exposure initially induced DNA hypomethylation, but prolonged exposure resulted in DNA hypermethylation (46). In an animal study, exposure to cadmium-and lead-contaminated soils caused tissue metal accumulation and epigenetic alterations (increased global DNA methylation levels) in rats (47). An inverse association between selenium and global DNA methylation was also shown *in vitro*, animal, and human studies (16). In the present study, we observed that increased blood lead or cadmium levels were associated with increasing 5mdC (%), while increased plasma selenium levels were associated with decreasing 5mdC (%). Our results were consistent with a Mexican birth cohort study (*n* = 144) which observed prenatal exposure to lead (measured from maternal blood specimens) was associated with elevated levels of lead exposure-related gene-specific DNA methylation among their children ages 11–18 (14). In a Taiwanese cross-sectional study that enrolled 738 participants of ages 12–30, Lin et al. observed that increased urinary concentrations of lead and cadmium were associated with 5mdC (%) when adjusting for covariates related to cardiovascular disease (48). In contrast to our observed association, in a Norwegian prospective birth cohort study (631 mother–child pair), Weyde et al. found that maternal blood selenium concentrations during pregnancy were positively associated with global DNA methylation (measured by 5-methylcytosine in percentage) both in mothers and newborns (49). Additionally, in a study that consisted of 202 Argentinian women with low levels of environmental cadmium exposure (median blood cadmium concentration: 0.36 μg/L), Hossian et al. found that urinary cadmium levels were inversely associated with global DNA methylation (measured by long interspersed nuclear element-1) (50). Previous studies in Bangladesh have supported that arsenic exposure is associated with global DNA methylation and epigenetic alterations can serve as biomarkers of arsenic toxicity (51, 52), inconsistent with our finding of a lack of association between total urinary arsenic and 5mdC (%). The urinary concentrations of arsenic found in both Bangladeshi studies (median urinary arsenic concentrations range from 137.3 to 201.5 μg/g creatinine for three cohorts) (51, 52) were extremely higher than those found in our study population, which may be one reason for the inconsistent findings. Differences in study designs and populations, exposure matrices, and indicators of global DNA methylation may lead to discrepant results when assessing the association between metal exposures and global DNA methylation. Further studies are needed to gain a better understanding of the association between exposure to metals and DNA methylation.

Our study showed that 5mdC (%) partially mediates the associations of blood lead or plasma selenium with eGFR. The observed mediated result may be reasonable because exposure to environmental toxic metals (e.g., lead) may induce inflammation through altered DNA methylation and, thus, reduce eGFR (53, 54). One study noted that specific DNA methylation levels in urine (SMTNL2 and G6PC), reflecting the proportion of exfoliated proximal tubule cells due to injury, were significantly associated with decreased eGFR (55). Additionally, in an epigenomic association study of kidney function and blood DNA methylation levels of 69 CpGs associated with declines in eGFR were identified, and most eGFR-related CpGs were also significantly associated with CKD and microalbuminuria (56). While not focusing on renal function, in a Taiwanese cross-sectional study of 738 young participants aged 12–30 (48), Lin et al. found that 5mdC (%) may mediate the association between urinary lead and carotid intima-media thickness, a marker of subclinical atherosclerosis. By reducing oxidative stress, it is possible that the association between selenium and increased eGFR may by decreasing global DNA methylation (57). A Chinese Kashin–Beck disease study found that selenium treatment decreased the methylation of O^6^-methylguanine-DNA methyltransferase, a DNA damage-repair gene that reduces chondrocyte damage by T-2 toxin (58). Also, selenium can significantly prevent tert-butyl hydroperoxide-induced chondrocyte apoptosis, mediated by reducing glutathione peroxidase 3 DNA methylation and increasing glutathione peroxidase 3 gene expression (59).

In this study, we found a significant synergic effect of blood cadmium and 5mdC (%) on CKD, possibly because exposure to cadmium may increase the production of reactive oxygen species and thereby cause oxidative stress. Excessive cellular reactive oxygen species can lead to damage to proteins, nucleic acids, lipids, membranes, and organelles, which are associated with various diseases, including CKD (60). In addition, DNA hypermethylation of specific genes that increase oxidative stress (57) and lead to renal fibrosis (61), together with high blood cadmium, increase the risk of CKD and this needs to be further explored. Our study also found that a significant multiplicative interaction effect of plasma selenium and 5mdC (%) on CKD. The reason may be that a high selenium state reduces inflammation and oxidative stress (62), followed by reducing DNA methylation lightening inflammation, and improving kidney function (54). Additionally, the present study identified a mediating role of global DNA methylation on the association between selenium and renal function based on the classical mediation approach that assumes linear association and no interaction among the variables. As our study also found suggestive evidence of interaction between selenium and 5mdC (%) on CKD, future prospective research with the more complex setting of exposure-mediator interactions (63) is warranted to accommodate in order to elucidate the relationship between metal exposures, global DNA methylation, and the risk of CKD.

Our study had several limitations. The case–control study findings should be interpreted cautiously in describing the temporal relationship between metal exposures, global DNA methylation, and eGFR, especially when interpreting the results of mediation analysis. Additionally, we calculated the interaction effects in the log-odds scale due to the case–control design. The OR estimates might be slightly biased as the high prevalence of CKD in the Taiwanese population (2). However, our results may inform future studies with a prospective design to enhance understanding of the underlying mechanism of DNA methylation in the etiology of exposure to metals and CKD. Second, plasma selenium, blood cadmium and lead, and total urinary arsenic concentrations were only assessed in urine and blood samples that were collected once, and so a possible misclassification error cannot be ruled out. However, these measured values may be reliable if all subjects maintained their usual lifestyle. Last, the statistical power may be limited because the number of samples in this study was relatively small; therefore, further work with a large sample size is warranted to provide adequate power.

## 5. Conclusion

In conclusion, our study found joint impacts of global DNA methylation marker, using 5mdC (%) as an indicator, with blood cadmium or plasma selenium on CKD, which supports the hypothesis of the adverse impacts of exposure to metals on CKD being modified by global DNA methylation. In addition, the present study provides suggestive evidence that global DNA methylation may partially mediate the association between exposure to metals (i.e., lead and selenium) and renal function measured as eGFR. This provides insights into the mechanisms of global DNA methylation for the metal exposures related to CKD.

## Data availability statement

The datasets presented in this article are not readily available because of privacy and ethical restrictions. Requests to access the datasets should be directed to the corresponding author.

## Ethics statement

The studies involving human participants were reviewed and approved by Institutional Review Board of Taipei Medical University (N201912103). The patients/participants provided their written informed consent to participate in this study.

## Author contributions

Y-MH, Y-CL, and H-SS partly contributed to the conception and design of the work. H-HC, Y-CL, and W-JC recruited the study subjects. H-LL has done the experiment. Y-MH, W-JC, and Y-LH contributed to the statistical analysis and analyzed the data. Y-MH wrote the original manuscript. W-JC, Y-LH, and S-LH reviewed and edited the manuscript. Y-CL performed the study design and executed the whole research plan. All authors contributed to the article and approved the submitted version.

## Funding

This study was supported by grants from the Ministry of Science and Technology of Taiwan (MOST 106-2314-B-038-066, MOST 107-2320-B-039-010, MOST 106-2314-B-002-235-MY3, MOST 107-2314-B-038-073, MOST 108-2314-B-038 -089, MOST 109-2314-B-038-081, MOST 109-2314-B-038-067, and MOST 110-2314-B-038-054). This study received no specific grant from any funding agency in the public, commercial, or not-for-profit sectors.

## Conflict of interest

The authors declare that the research was conducted in the absence of any commercial or financial relationships that could be construed as a potential conflict of interest.

## Publisher’s note

All claims expressed in this article are solely those of the authors and do not necessarily represent those of their affiliated organizations, or those of the publisher, the editors and the reviewers. Any product that may be evaluated in this article, or claim that may be made by its manufacturer, is not guaranteed or endorsed by the publisher.
