# Supplementary material for: Global DNA methylation and the association between metal exposure and chronic kidney disease
Source: Front Public Health. 2023 May 25;11:1104692. doi: 10.3389/fpubh.2023.1104692 (PMC10248129; doi:10.3389/fpubh.2023.1104692)
Supplement: Supplementary file 1 [file Table_1.DOCX]

Supplementary Material

**Table S1**. The validity and reliability of urinary arsenic species, plasma selenium, and red blood cell lead and cadmium.

| Metals or metalloids | Method | Detection limit (μg/L) | Recovery rate | SRM | CV% |
| --- | --- | --- | --- | --- | --- |
| Plasma selenium | Inductively coupled plasma mass spectrometry | 0.193 |  | SRMs (Seronorm Trace Elements Whole Blood Label II (SERO AS, Norway) contained 112 ± 46 mg/L of selenium, in our system 118.7 ± 11.1 mg/L (n = 7) | 9.8% |
| Red blood cell lead | Inductively coupled plasma mass spectrometry | 0.32 |  | SRMs (Seronorm Trace Elements Whole Blood L-2 (Lot 1103129)) certificate value 310.0 μg/L (range 186.0–434.0 μg/L), in our system 329.0 ± 17.0 μg/L | <10% |
| Red blood cell cadmium | Inductively coupled plasma mass spectrometry | 0.07 |  | SRMs (Seronorm Trace Elements Whole Blood L-2 (Lot 1103129)) certificate value 5.8 μg/L (range: 5.4–6.2 μg/L), in our system 6.1 ± 0.5 μg/L | <10% |
| Arsenite (As^III^) | High-performance liquid chromatography-hydride generator-atomic absorption spectrometry | 0.02 | 93.8–102.2% | SRM (National Institute of Standards and Technology (NIST, Gaithersburg, MD) 2670 certificate value 480 ± 100 μg/L inorganic arsenic, in our system 507 ± 17 μg/L (n = 4) | <10% |
| Arsenate (As^V^) |  | 0.10 |  |  |  |
| Monomethylarsonic acid (MMA^V^) |  | 0.07 |  |  |  |
| Dimethylarsinic acid (DMA^V^) |  | 0.06 |  |  |  |
